# Data driven models on load forecasting: Case study Algeria

**DOI:** 10.1016/j.dib.2023.109854

**Published:** 2023-11-26

**Authors:** Rania Farah, Brahim Farou, Zineddine Kouahla, Hamid Seridi

**Affiliations:** Department of Computer Science, LabStic Laboratory, University 8 May 1945, Guelma, Algeria

**Keywords:** Energy, Electricity, Machine learning, Statistical techniques, Accurate predictions

## Abstract

Databases are indispensable in many areas. This historical database contains a wealth of information, such as the energy. Energy is a vital resource in our daily lives, for both residential and industrial users. However, determining the exact amount of energy required to satisfy society's demands remains a difficult question. The only way for the moment to solve this problem is to create models using historical data. The Algerian provider has given this data in order to prepare for consumption according to the proposed models. This article focuses on hourly consumption data collected between 2008 and 2020, carefully collected to enable efficient energy modelling and enhance forecasting. This data serves as the basis for the development of statistical, mathematical and predictive models based on machine learning principles. To achieve this, an advanced analysis of the dataset is applied, using statistical techniques and concepts that will provide valuable insights and statistical knowledge, enabling them to make accurate predictions.

Specifications Table

**Table:** 

Subject	Energy
Specific subject area	Energy forecasting using Deep learning models.
Data format	Raw, Analysed, Excel
Type of data	Table (raw data)
Data collection	Sonelgaz has been equipped with several power centers that are interconnected by a local computer network which allows the measurement of each of these power centers and everything is summarized by a SCADA (Supervisory Control and Data Acquisition) software. These collected data are transferred in a Excel sheet.
Data source location	Algeria (Africa)
Data accessibility	The data is available in the Mendeley Data repository : https://data.mendeley.com/datasets/z5×2d3mhw7/1 DOI:10.17632/z5×2d3mhw7.1

## 1 Value of the Data

• The data set can be used as the most important information for modelling accurate forecasting models.
• The data set can be used to study energy consumption patterns and systems in all Mediterranean and other countries.
• The data can be used as a tool to explain how energy can be managed and used for these systems.
• The data are the evolution of the consumption during 12 years, and can be used to study the evolution of the society and other economic models.
• Forecasting models, for Short, medium and long term.

## 2 Data Description

In this study, the national electricity consumption of Algeria is used, a country with a surface area of 2,381,741 km², making it the tenth largest nation in the world by total surface area, and the largest nation in Africa and the Arab world. Algeria is located in the Maghreb region of North Africa, stretching 1,622 km from the Mediterranean coast to the great Sahara Desert. The electricity consumption data comes from Sonelgaz, Algeria's leading electricity producer and supplier. The data were recorded every hour over a 12-year period, from January 2008 to January 2020, which means there is 4406 samples and 24 characteristics. These data were subjected to statistical analysis, including daily, seasonal and annual correlation analysis.

[1] Showed that the electrical load is still increasing, and that Algerians consume more electricity in summer than in winter.

### 2.1 Data Analysis

The presented database contains 12 years of electricity consumption. Different operation must be done to understand the evolution of the data. While the mean provides an approximation of the central tendency in Megawatt (MW), the maximum and lowest values provide information about the upper and lower boundaries of the data. Also, it is helpful to calculate the standard deviation (Std), when evaluating the variability of the data, which shows the dispersion of values around the mean. Table 1 summarizes the most important data of the 24 hours of the day (max, min, mean and Std)

**Table 1:** Important information of the data.

Hours	Max	Min	Mean	Std
1h	11956	2842	5478.772913	1689.245760
2h	11422	2618	5182.804729	1611.402635
3h	11040	2590	5015.457269	1529.642495
4h	10632	2513	4935.584473	1471.294068
5h	10384	2541	4897.559471	1393.384042
6h	10010	2625	4930.873883	1289.739058
7h	9382	2603	5008.771185	1190.720152
8h	9049	2510	5213.010709	1172.261639
9h	9711	2644	5558.865317	1222.396369
10h	10282	2771	5774.921059	1288.680800
11h	10449	2754	5874.434619	1369.334908
12h	11120	2747	5962.521002	1484.623066
13h	12231	2714	6010.496624	1645.176994
14h	13000	2671	6093.044245	1817.227662
15h	13222	2604	6083.870347	1883.545869
16h	12976	2574	6042.665642	1888.002975
17h	12706	2614	5973.628268	1817.690240
18h	11752	2707	5943.532476	1596.218119
19h	10614	3157	6235.312066	1427.824761
20h	11000	3508	6612.912514	1319.148188
21h	11847	3924	6878.772534	1402.947153
22h	12374	3181	6757.359866	1522.040151
23h	12407	2856	6406.057225	1631.781861
24h	12352	2434	5914.433807	1714.623077

The coefficient of variation (CV) is the percentage ratio of the standard deviation to the mean Eq. (1).

(1) CF=STD/Mean*100

**Fig. 1:**
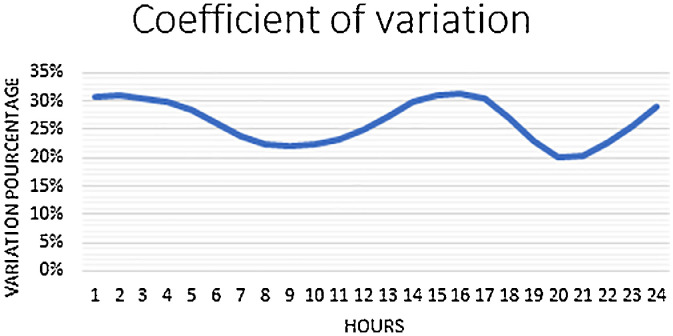
Coefficient of variation curve.

A high value of the CV indicates a higher level of dispersion or fluctuation around the mean. In the context of electrical energy consumption, a higher CV suggests greater variability in consumption patterns. However, it is important to note that the curve representing the percentage variation does not exceed 31% as shown in Fig. 1 and the mean of CV is about 26%. This indicates that the database has a relatively homogeneous distribution of values, with a moderate level of fluctuation around the mean.

## 3 Experimental Design, Materials and Methods

The data is the essential material to build a model. Data analysis and visualization can be used to explore and discover patterns, trends, correlations and anomalies in the data in real-time [2].

Accurate load forecasting for the electricity supplier is an essential operation, the supplier then, can make an economic and a safe distribution of this energy [3].

### 3.1 Correlation

**Fig. 2:**
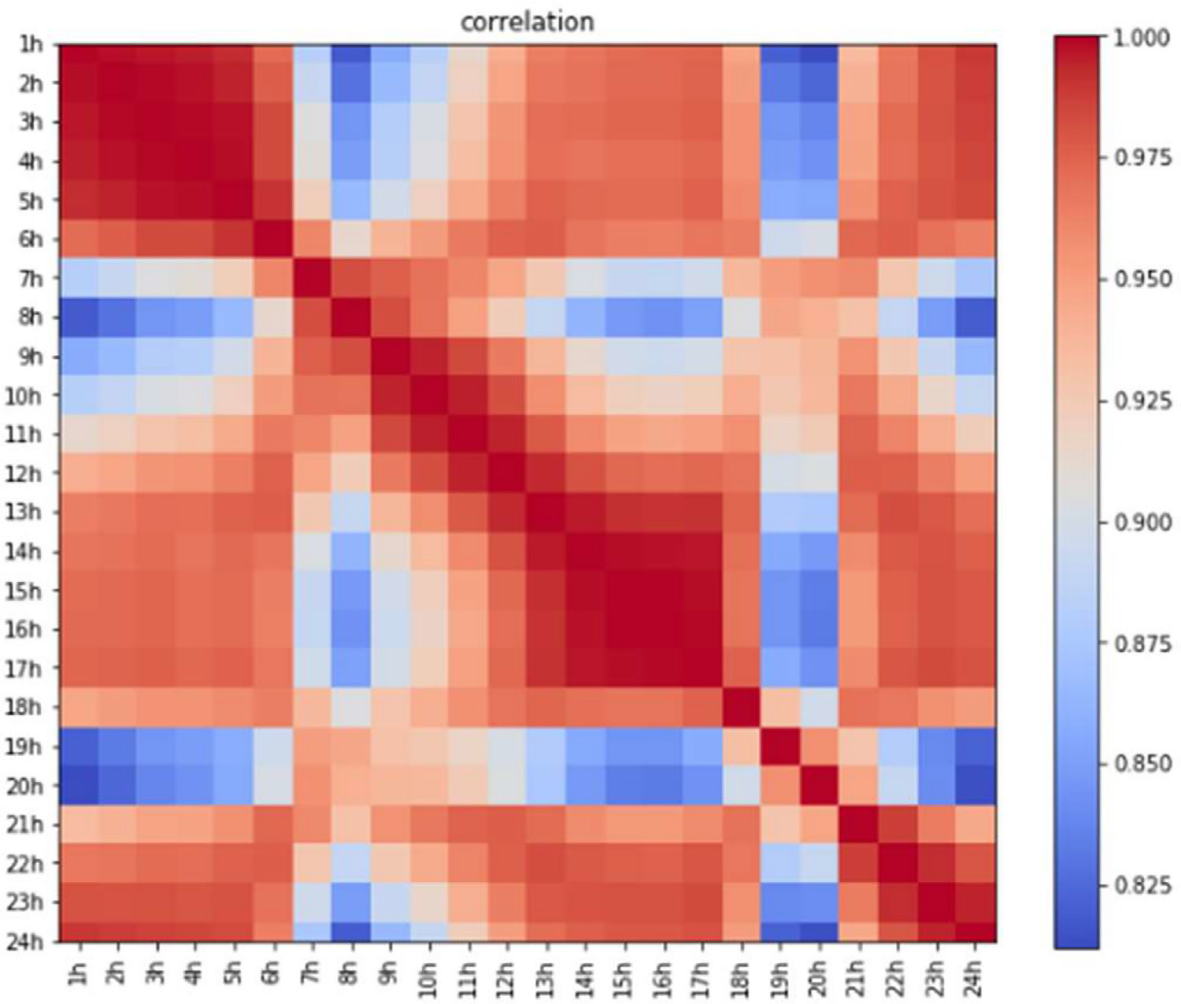
Correlation of the data.

Correlation can provide information on the direction (positive or negative) and strength of the relationship between variables. As shown in Fig. 2 there is a very strong correlation between some hours of the days [4].

It is important to note that a strong correlation may imply the variables correlated with each other belong to the same time period (day, night, beginning of the day...etc.). All of which can influence electricity consumption.

### 3.2 Clustering

The clustering operation with K-means method shows different groups [5] that are generally represented by the seasons or different behaviors.

**Fig. 3:**
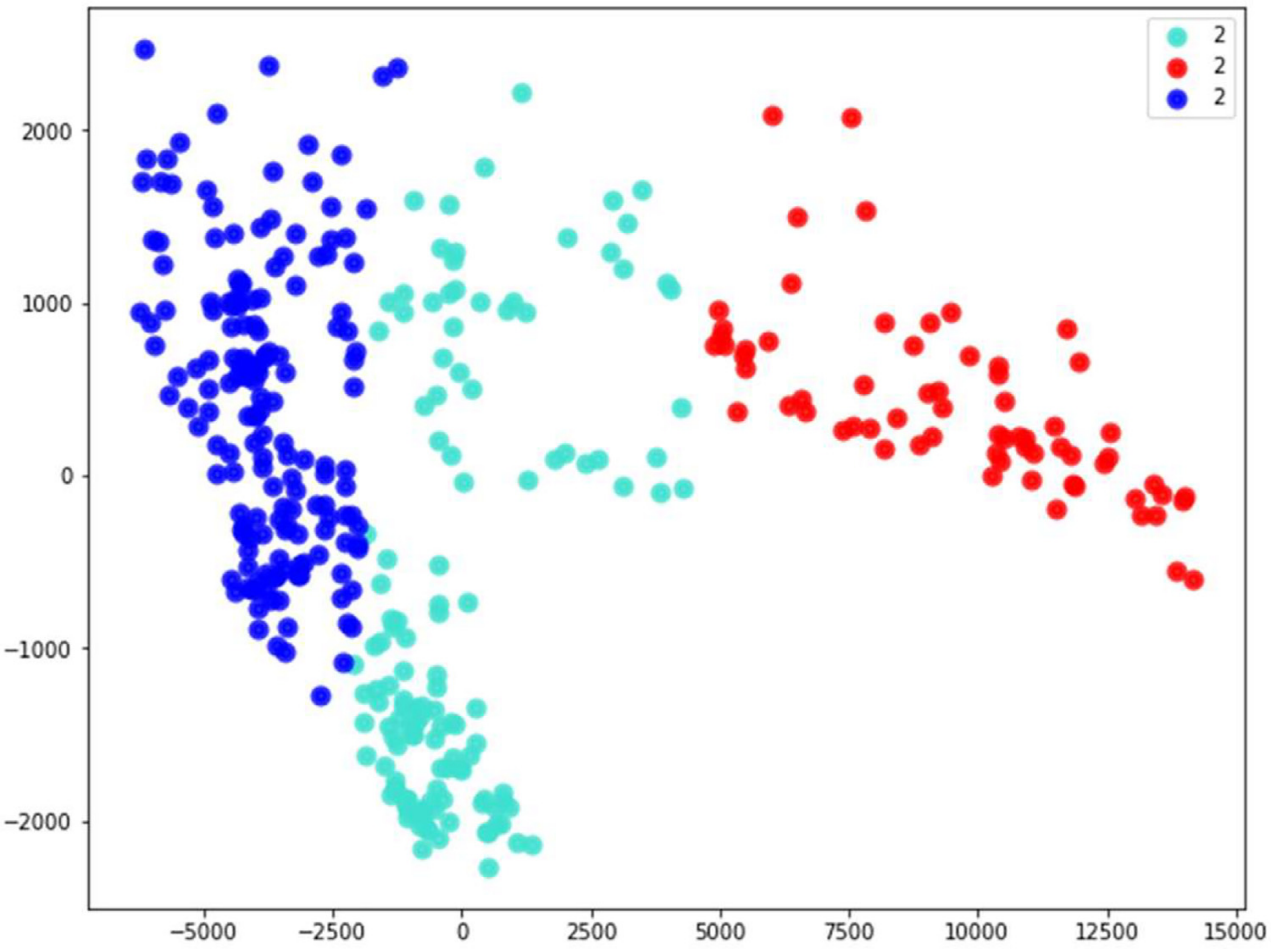
Cluster of the year 2015.

Fig. 3 represent clustering operation of the year 2015 which shows three clusters. These clusters represent the different seasons of the year note that: spring and autumn are represented by the same cluster due to their similar behavior.

**Fig. 4:**
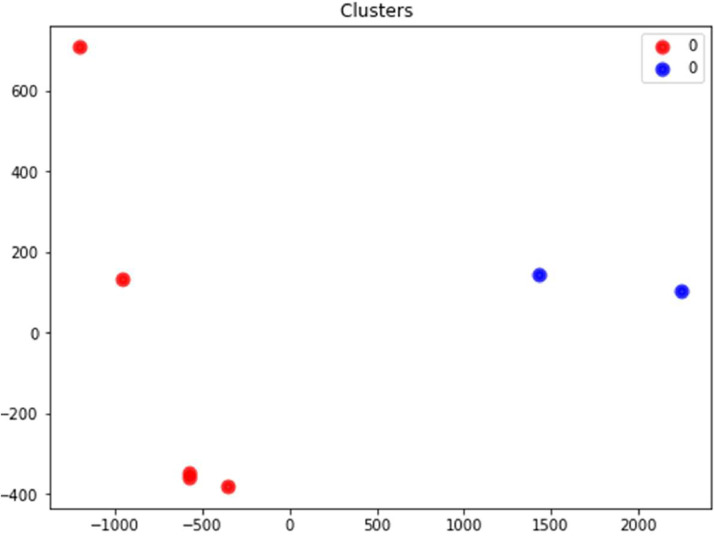
Cluster of a week in winter 2015.

The Fig. 4 represents the three clusters of one week in winter of the year 2015. This Figure clearly shows that there is three clusters that represent the workdays, weekends and before the workday.

In Algeria, the weekend are Friday and Saturday, which means that these two days are grouped together in a single cluster. Saturday, on the other hand, is regarded as a somewhat special day, generally considered as a weekend for the civil service, although other companies may work on it. As for working days, they are grouped together in another separate cluster, reflecting their common consumption patterns [6].

With the knowledge of this clustering method, it is possible to understand the various consumption patterns that are seen during the week and on the weekends, which is helpful for energy management. To better understand these habits, a profile study is required.

### 3.3 Profile

**Fig. 5:**
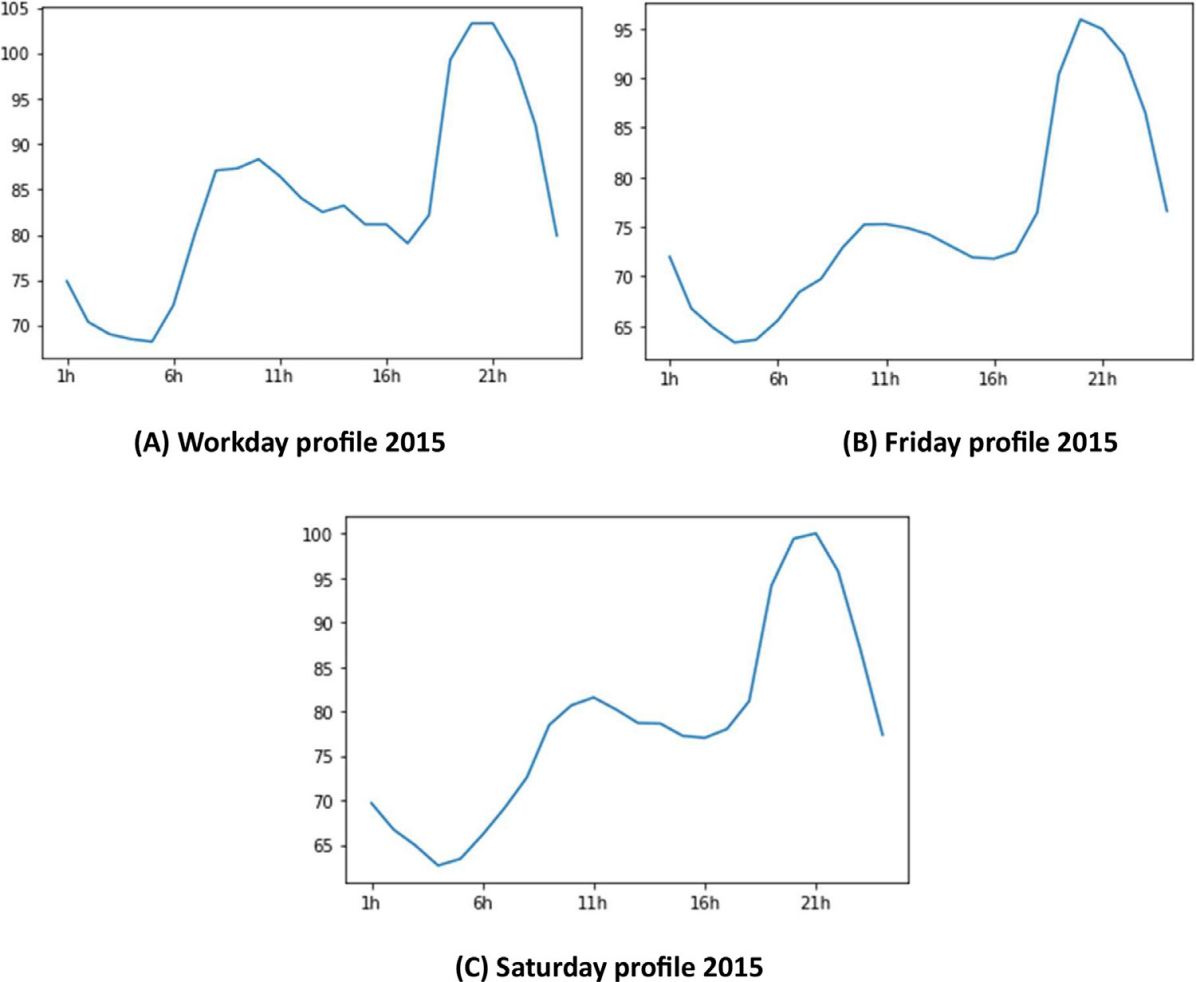
Different days profiles.

Fig. 5 represents different profiles, working days profile (A), weekend profiles (B), and Saturday profile (C). Since the profiles are different, these information will make it possible to study this dataset for modeling and forecasting purposes.

### 3.4 Dispersion of the data

The standard deviation of energy consumption per hour in this instance show the fluctuation in energy usage throughout the day.

**Fig. 6:**
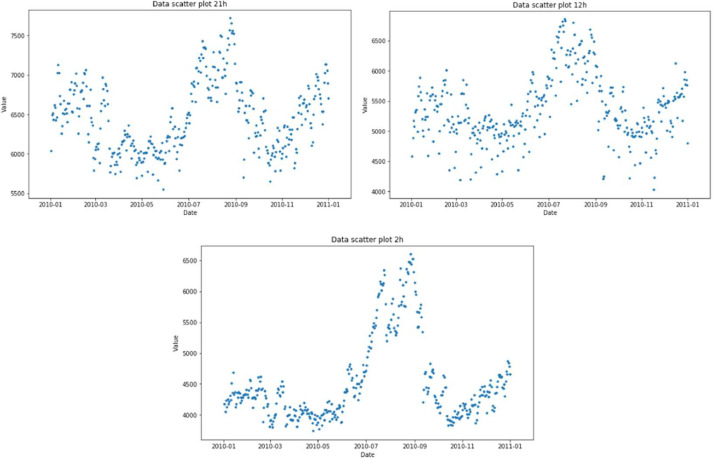
Dispersion of 2a.m, 12a.m and 9p.m.

The Fig. 6 shows that there are periods of high consumption and others of low consumption, but the overall trend over the years is rising.

The distribution in Fig. 6 shows that the consumption peaks in August, which is in summer, as a result of the extreme heat and the use of air conditioners, which likely contributes to the significant increase in power demand during this season.

**Fig. 7:**
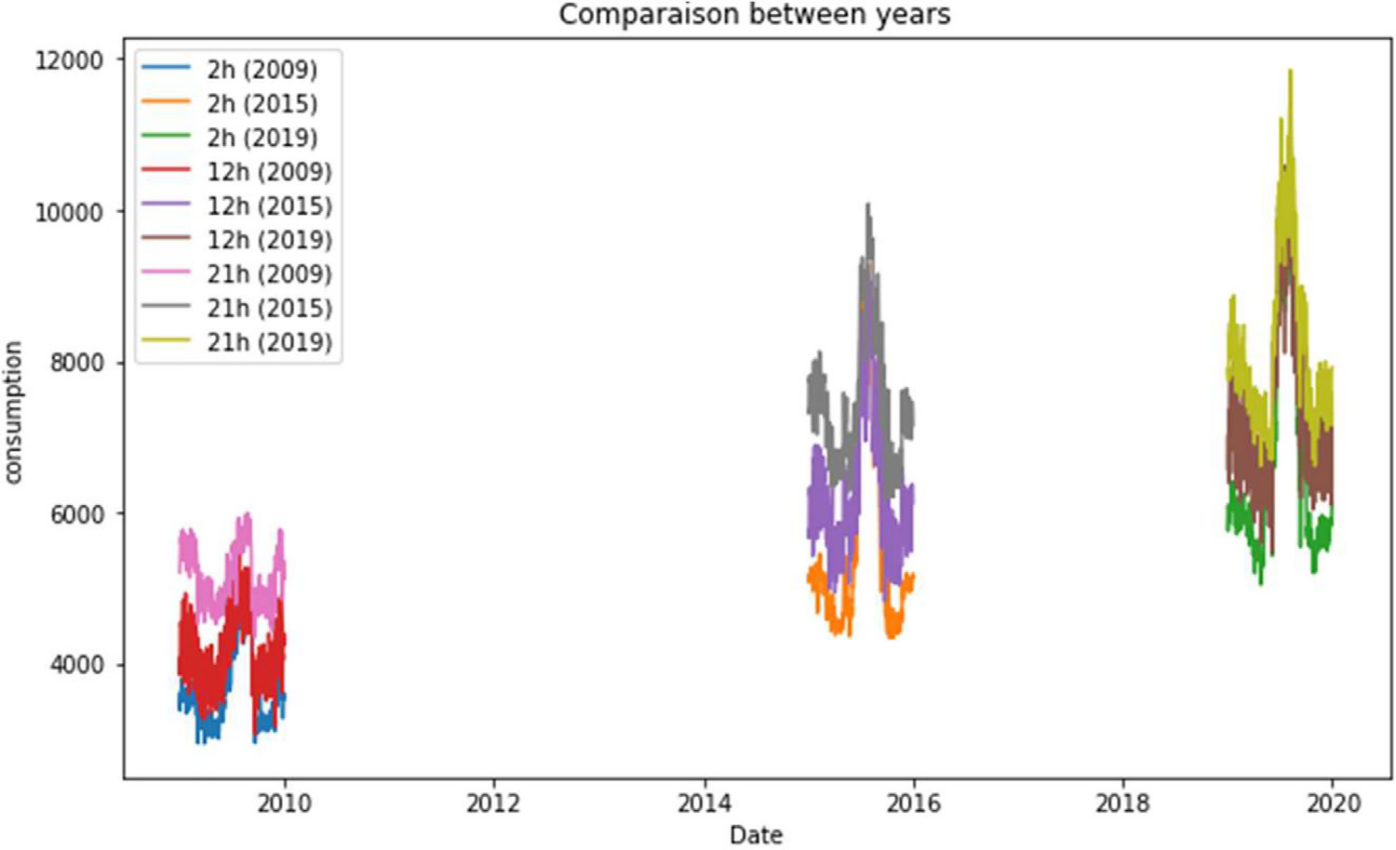
Comparison of different hours of the day in different years.

Fig. 7 shows low consumption at night and high consumption during the day, due to human activities and the trend is rising during the years.

### 3.5 *Trend*

The study of frequency, is also essential, focuses on the regularity of time samples in a time series. It represents the time interval, which in the case of this study is one day.

**Fig. 8:**
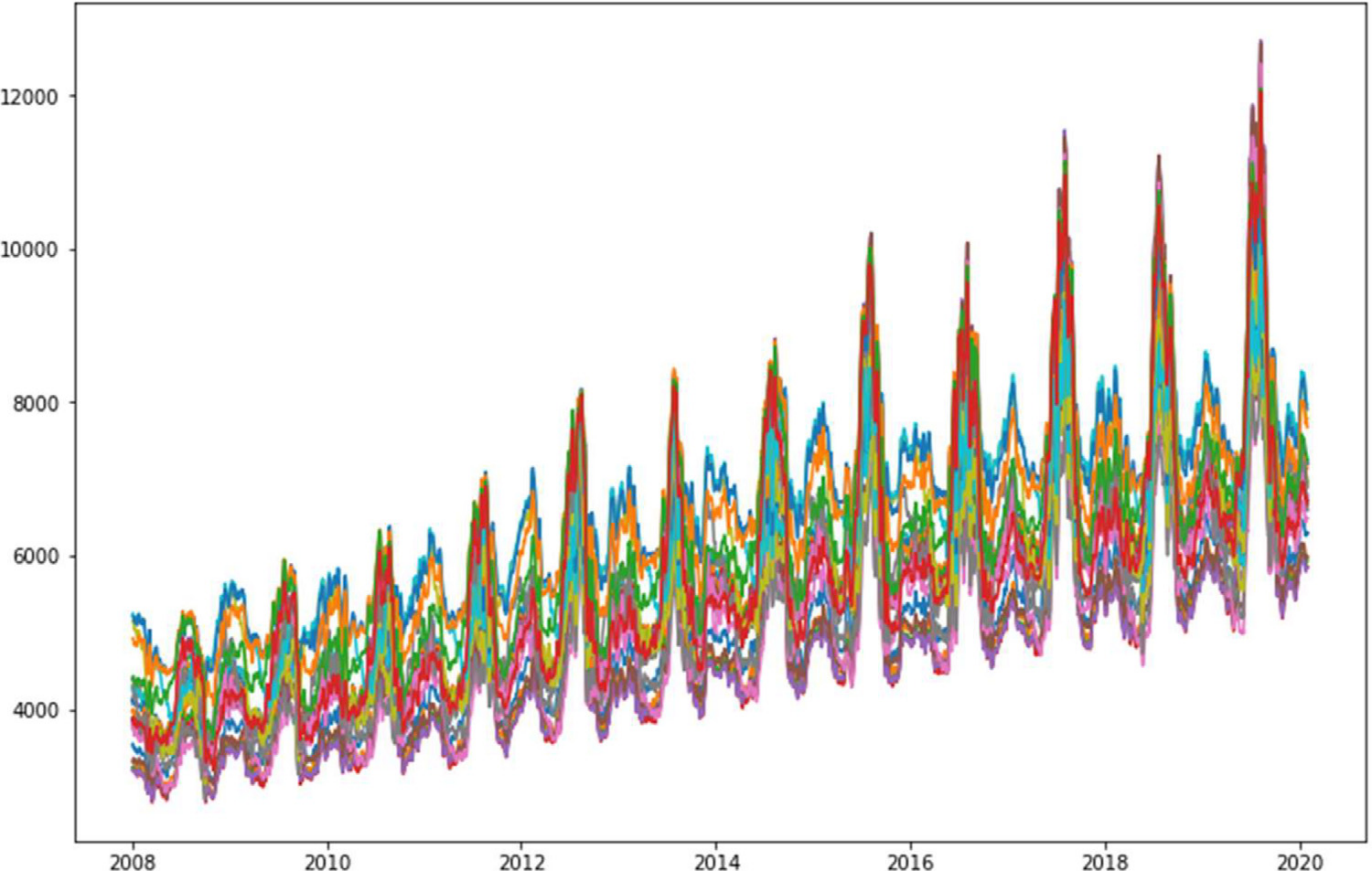
Trend cycle of the Date for each hour.

Frequency enables to identify any recurrent or seasonal trends in the time series as in this study the electrical consumption in Fig. 8. The consumption is evolving on various time scales, such as daily, weekly, monthly, or annual changes, which can be understood by determining the periodicity of trends.

Frequency is also important in forecasting and modeling time series [6]. By knowing the frequency of seasonal or cyclical patterns, we can choose appropriate forecasting models that capture these temporal patterns and improve forecasting [7].

Visually, Fig. 8 shows a repeating cycle with an amplitude that increases over the years. To do this, the periodicity of the repeating cycle needs to be determined over the year. In this case, a periodicity is found for one day, 217 and 273 days.

As the 217 and 273 day's (appromximately 9 months) frequencies may represent particular seasons and seasonal cycles, the 1 day frequency represent the different events happaning in 24 hours.

**Fig. 9:**
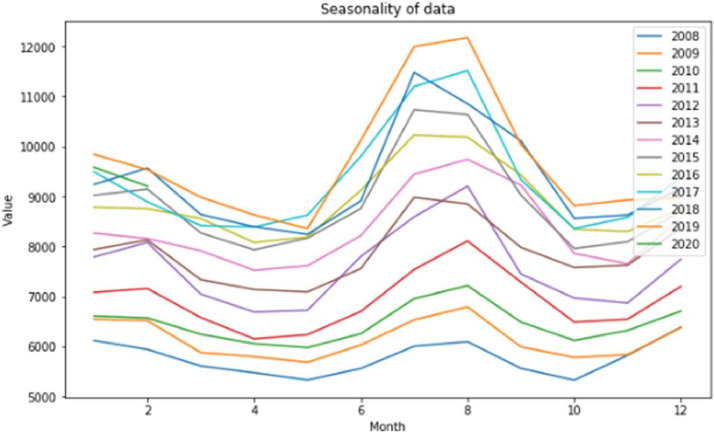
Profile for each year.

The Fig. 9 shows that the trend of a season, represented by its profile, is the same every year, the difference is in the amplitude. The peak of the consumption is found in the summer season, where it can be very hot.

One possible experiment with this database is forecasting with Several deep neural networks architectures to solve the problem of electric load forecasting for different horizons (short, medium and long term) [8]. A model for every cluster [9], or a global model, that can catch the evolution of the demand for each day, for a season or for a year.

There are some examples of experimentations in Table 2 which suggest that both the Convolutional Neural Network (CNN) model and the linear regression model present a score error very low with the metrics Root Mean Square Error (RMSE) and Mean Absolute Error (MAE). This can be considered as an excellent perspective.

**Table 2:** Example of the experimentations (Global model for Short term load forecasting).

Models	RMSE	MAE
Linear Regression	2.921642e-12	3.931761e-14
Decision tree	1.686524e+02	2.046785e+00
CNN	44.08	31.189

## Limitations

None.

## Ethics Statement

The authors confirm that the current work does not involve human subjects, animal experiments, or any data collected from social media platforms.

## CRediT authorship contribution statement

**Rania Farah:** Conceptualization, Methodology, Writing – original draft, Visualization. **Brahim Farou:** Conceptualization, Methodology, Writing – review & editing. **Zineddine Kouahla:** Conceptualization, Methodology, Software, Validation. **Hamid Seridi:** Writing – review & editing, Supervision.

## Data Availability

Load Consumption Data Algeria (Original data) (Mendeley Data)
